# Reducing Malaria-Related Under-Five Mortality in Nigeria Through Scalable Prevention and Treatment Strategies

**DOI:** 10.7759/cureus.113652

**Published:** 2026-07-30

**Authors:** Andre C Christie, Jessica Pelletier

**Affiliations:** 1 School of Public Health, New York University, New York, USA; 2 Department of Medicine, Trinity School of Medicine, Warner Robins, USA; 3 Department of Emergency Medicine, University of Missouri-Columbia, Columbia, USA

**Keywords:** community health workers, malaria, nigeria, rts, seasonal malaria chemoprevention, s vaccine, under-five mortality

## Abstract

Malaria remains a major health challenge in Nigeria, particularly among children under five years of age. Although efforts to control the disease have expanded, important implementation challenges remain. This editorial reviews the major drivers of under-five mortality due to malaria, including low coverage of malaria prevention interventions, poor access to prompt diagnosis and appropriate treatment, and persistent socioeconomic and health system challenges. It also identifies gaps in health services, the supply chain, and human resources that hamper the implementation of current malaria control strategies. The editorial recommends three feasible and scalable interventions: scaling up seasonal malaria chemoprevention, integrating malaria vaccination into routine childhood immunization services, and improving community-based case management. These recommendations aim to complement the current health system and improve access to effective malaria prevention and treatment. Their implementation could reduce malaria-related deaths among children under five and contribute to Nigeria’s progress toward malaria control and elimination.

## Editorial

Introduction

Infections resulting from malaria continue to account for most malaria-related deaths among children under five years of age in Nigeria. Malaria remains a major burden in Nigeria and, indeed, worldwide, as the country accounted for approximately 27% of the world’s malaria cases and 30.9% of global malaria deaths in 2023 [1,2]. In the same year, 184,689 people in Nigeria lost their lives due to malaria [2]. Children under five years of age are disproportionately affected, accounting for 39.3% of global malaria deaths in this age group in 2023 [2]. Of the 598,000 malaria deaths reported globally that year, 73.7% occurred among children under five, corresponding to approximately 140,000 deaths in Nigeria [2].

**Figure 1:**
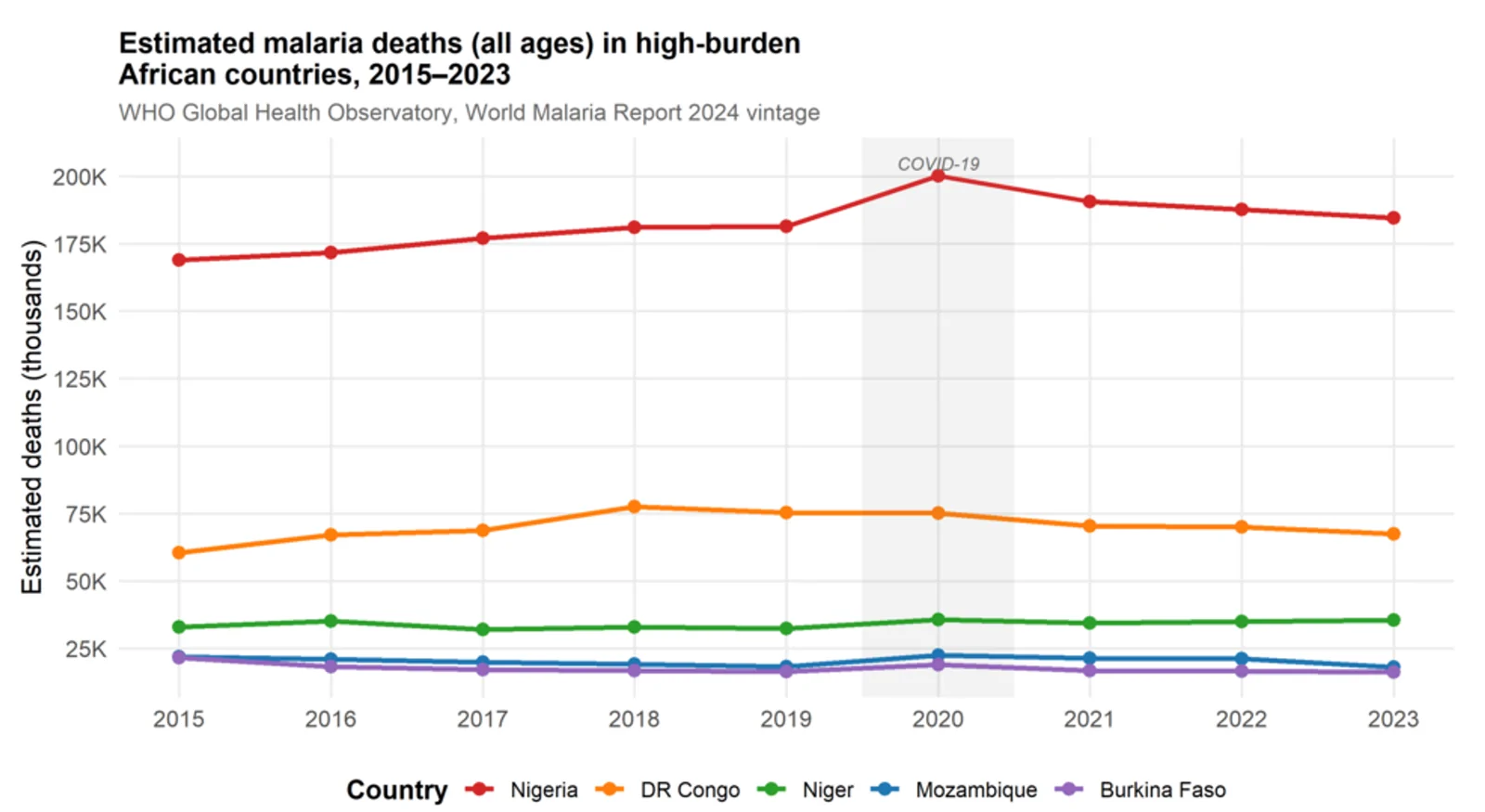
Estimated malaria deaths (all ages) in high-burden African countries, 2015-2023 Approximately 76% of malaria deaths in the WHO African Region occur among children under five years of age. Adapted from data reported in the World Malaria Report 2024: Addressing Inequity in the Global Malaria Response [2]. This figure was created using the R statistical programming language (version 4.6.0; R Foundation for Statistical Computing, Vienna, Austria) and the ggplot2 data visualization package (version 3.5.x; Posit PBC, Boston, MA, USA).

The scale of malaria mortality in Nigeria is even more evident when compared with other high-burden African countries. The number of malaria deaths in Nigeria in 2023 was nearly three times that in the Democratic Republic of the Congo (67,464 deaths), the country with the second-highest number of malaria deaths in Africa (Figure 1) [2]. Malaria deaths totaled 35,381 in Niger, 17,875 in Mozambique, and 16,146 in Burkina Faso. These figures are substantially lower, despite similarly high levels of malaria transmission in these countries [2]. The number of malaria deaths in Nigeria consistently exceeded 169,000 between 2015 and 2023, with a marked increase in 2020, likely due to disruptions caused by the COVID-19 pandemic [2].

**Figure 2:**
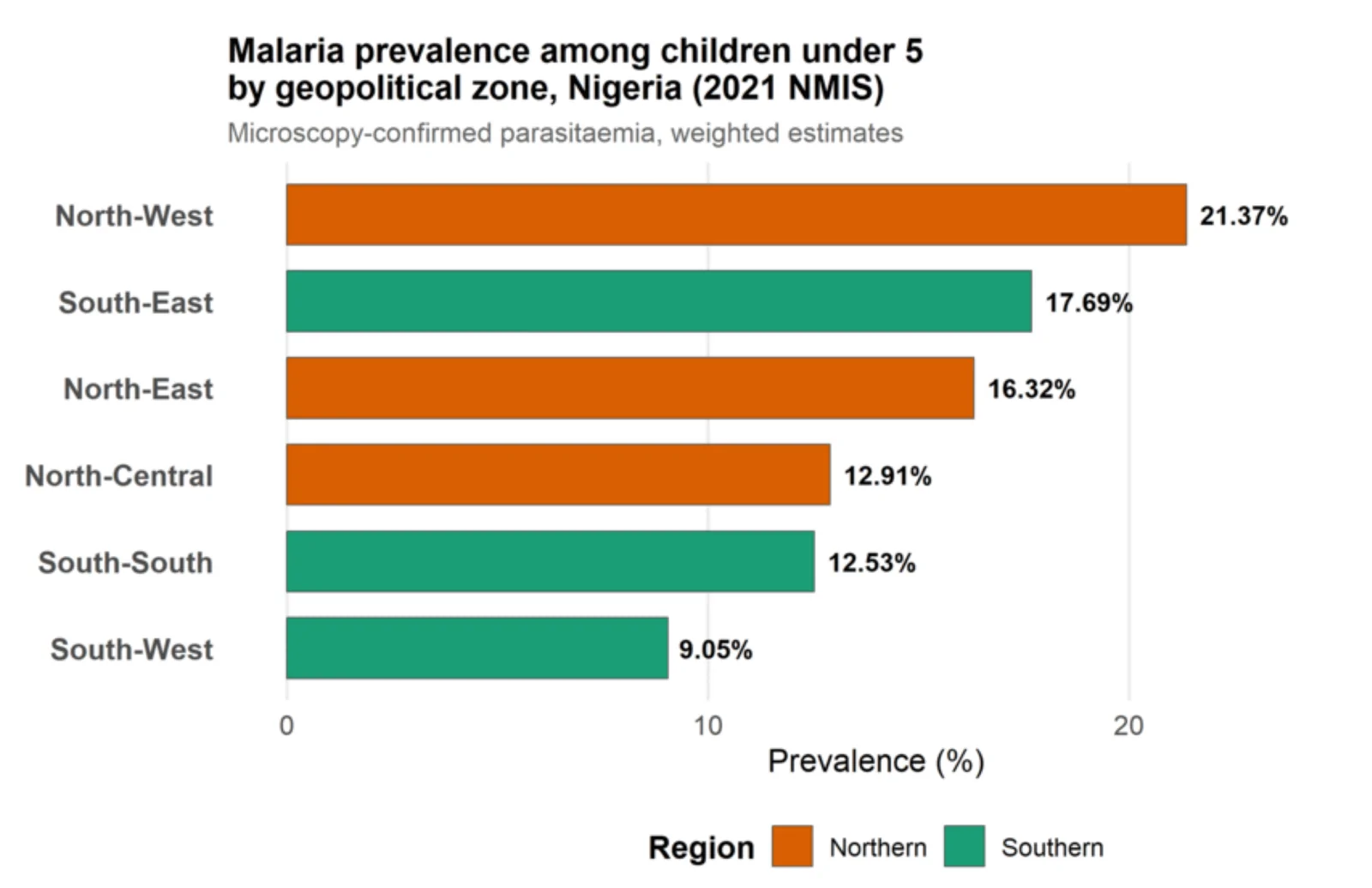
Malaria prevalence among children under five by geopolitical zone N = 10,645 children aged 6-59 months NMIS, Nigeria Malaria Indicator Survey Adapted from data reported by Isiko et al. (2024) [5] This figure was created using the R statistical programming language (version 4.6.0; R Foundation for Statistical Computing, Vienna, Austria) and the ggplot2 data visualization package (version 3.5.x; Posit PBC, Boston, MA, USA).

Poverty and geography further increase these vulnerabilities in Nigeria. Approximately 40% of the Nigerian population lives below the poverty line, and the prevalence of malaria among children under five years of age varies by geographic region [3,4]. Data from the 2021 Nigeria Malaria Indicator Survey (NMIS) show that among children aged 6-59 months, the highest malaria prevalence was recorded in the North-West Zone (21.4%), whereas the lowest was recorded in the South-West Zone (9.1%) (Figure 2) [3,5]. The South-East (17.7%) and North-East (16.3%) Zones had the second-highest prevalence, followed by the North-Central (12.9%) and South-South (12.5%) Zones [5]. All three northern zones had prevalence rates above the national average, which is associated with higher poverty levels and limited access to healthcare services. In 2021, only 41% of Nigerian children under five years of age used insecticide-treated nets (ITNs), and ITN use was significantly lower in poor rural areas (Figure 2) [3]. Limited access to healthcare facilities prevents many febrile children from receiving timely diagnosis and treatment. Overall, these data indicate that children living in poor rural areas with inadequate housing and limited access to preventive measures bear the greatest burden of malaria.

Discussion

Malaria deaths among children under five years of age in Nigeria can be traced to four interrelated causes: (1) insufficient use of indoor residual spraying (IRS) and seasonal malaria chemoprevention (SMC); (2) inadequate utilization of health care facilities; (3) socioeconomic factors such as poverty, low levels of maternal education, and poorly designed houses that expose children to mosquitoes; and (4) environmental and climatic conditions, especially the extended rainy season, which promotes increased mosquito breeding and disease transmission [2,4]. These factors interact with one another, and their cumulative effect results in very high malaria mortality rates among Nigerian children.

Inadequate Access to Prevention Measures

Several effective malaria prevention interventions are available but have not been uniformly implemented across the country, particularly in areas with a high disease burden. Long-lasting ITNs are one of the most fundamental measures for preventing malaria, but even after large-scale distribution, ITN coverage among children does not meet the national target [4]. SMC has been shown to be an effective intervention for preventing clinical and severe malaria episodes in children; however, it has not been fully implemented in the northern states with the highest disease burden [6,7]. Similarly, IRS has not been fully utilized and has failed to reach many areas that could benefit from it [4]. Each of these coverage gaps leaves children vulnerable to preventable infection, and the gaps are greatest in the regions that already carry the highest disease burden (Figure 2).

Limited Healthcare Access

Timely diagnosis and treatment are challenging for many Nigerian children. In many rural and remote communities, hospitals lack adequate supplies and equipment. As a result, parents of sick children face barriers such as treatment costs and transportation, delaying hospital visits until their child becomes seriously ill [1]. Moreover, stockouts of rapid diagnostic tests and artemisinin-based combination therapies (ACTs) further impede effective case management, even in accessible health care facilities [1]. The importance of early detection cannot be overstated, as severe malaria is associated with higher mortality even when treated [2].

Socioeconomic Factors

Social and geographic disparities contribute to the uneven distribution of malaria-related morbidity and mortality. Inadequate housing and poor sanitation expose people in low socioeconomic communities to environments that favor mosquito breeding [3,8]. Moreover, people in these communities often have limited resources to seek preventive care and treatment [4,6]. As shown by the regional differences presented in Figure 2, the northern zones, which are characterized by higher poverty levels, have higher malaria prevalence and mortality rates than the southern zones [3-5]. Maternal education is another important determinant of malaria risk. Children born to mothers without formal education are more likely to become infected with malaria parasites than children whose mothers have received formal education [5].

Environmental and Climatic Factors

Environmental factors facilitate year-round malaria transmission in many parts of Nigeria while increasing transmission peaks during specific seasons. In northern Nigeria, seasonal rains, hot weather, and poor drainage create ideal breeding conditions for *Anopheles* mosquitoes, leading to increased disease transmission during and after the rainy season [6,8]. Environmental changes, including climate change, rapid urbanization, and environmental mismanagement, have also contributed to increased mosquito breeding in many areas.

Future Directions

Interventions to reduce malaria mortality among Nigerian children have been available for many years. The primary challenge lies in the health system’s ability to distribute these interventions effectively amid shortages of skilled health personnel, poor logistical management, inadequate supervision and monitoring, and heavy dependence on international funding [4,6]. Three evidence-based interventions could be scaled up immediately: SMC, malaria vaccination, and community-based case management [1,2].

SMC is one of the most effective interventions for protecting children in regions with highly seasonal malaria transmission. Several studies conducted across Africa indicate that SMC can reduce clinical malaria cases by as much as 75% and prevent severe malaria in children under five years of age [7]. Expanding the reach of SMC should remain a national priority in the high-burden northern regions, which have the highest malaria prevalence, as shown in Figure 2 [6].

Remarkable progress has also been made in malaria vaccination. Currently, the World Health Organization recommends two vaccines for reducing malaria in children: the RTS,S/AS01 (Mosquirix) vaccine, recommended since 2021, and the R21/Matrix-M vaccine, recommended in October 2023 [9,10]. During its phase 3 trials conducted in four African countries, the R21/Matrix-M vaccine demonstrated 75% efficacy against clinical malaria within 12 months at seasonal transmission sites and 79% efficacy among children aged 5-17 months [10]. Nigeria received its first one million doses of the R21/Matrix-M vaccine in October 2024. Vaccine deployment began in December 2024 in Kebbi and Bayelsa States for children aged 5-15 months [11]. The campaign in Kebbi alone is expected to immunize 179,542 children using the four-dose vaccination schedule [11]. To ensure successful implementation, malaria vaccination should be integrated into the routine immunization program, cold chain systems should be strengthened, health care personnel should be trained, and awareness campaigns should be conducted in rural communities [6,9].

Strengthening community-based case management is another essential priority. The use of community health workers (CHWs) is a feasible strategy for bringing malaria diagnosis and treatment closer to children in areas where formal health care infrastructure is limited or absent. Through integrated community case management (iCCM), CHWs can perform diagnostic testing, provide timely treatment with ACTs, deliver health education, and make referrals at the household level [1,12]. According to the WHO Rapid Access Expansion (RAcE) project, which included Nigeria as one of the implementation sites, the introduction of iCCM reduced under-five mortality by approximately 10%, with nearly 75% of the lives saved attributable to CHW-delivered care rather than facility-based health care [12]. To ensure the sustainability of these programs, ongoing support is needed in the form of training, supervision, and a reliable supply of essential commodities.

None of these interventions will achieve their full potential if implemented in isolation. Their effectiveness depends on broader efforts to strengthen the health system, expand the health workforce, and secure more sustainable financing. As Figure 1 illustrates, Nigeria’s malaria death toll has remained persistently and disproportionately high for nearly a decade. A coordinated strategy that combines prevention, vaccination, and early treatment offers the greatest opportunity to reverse this trend [1,2].

Conclusions

Malaria continues to be one of the leading causes of mortality among children in Nigeria. The most vulnerable groups are children under five years of age living in poverty and in rural areas, particularly in the northern regions. Despite the ambitious objectives outlined in the country’s malaria strategy, there remains a need to scale up key malaria control measures. SMC should be expanded in the high-burden northern regions. Malaria vaccination with the R21/Matrix-M and RTS,S vaccines should be expanded nationwide. Community-based case management through iCCM could help address these challenges.
